# The burden of anemia among Chinese HIV-infected patients following the initiation of antiretroviral therapy in the treat-all era: a nationwide cohort study

**DOI:** 10.1186/s12879-023-08675-1

**Published:** 2023-10-19

**Authors:** Lai Wei, Yan Zhao, Xiumin Gan, Decai Zhao, Yasong Wu, Zhihui Dou, Ye Ma

**Affiliations:** Division of Treatment and Care, National Center for AIDS/STD Control and Prevention, Chinese Center for Disease Control and Prevention, No. 155, Changbai Road, Changping District, Beijing, China

**Keywords:** HIV, Anemia, Antiretroviral therapy, Mortality

## Abstract

**Background:**

To assess the prevalence of anemia before and after antiretroviral therapy (ART) initiation and to identify impact of anemia on mortality among HIV-infected patients in China during the Treat-All era.

**Methods:**

All HIV-infected patients who newly initiated ART between January 1, 2017 and December 31, 2020 were enrolled and followed up to December 31, 2021 in China. We analyzed the prevalence of anemia before and after ART initiation. Generalized estimating equations were fitted to determine factors associated with anemia after ART. Time-dependent cox proportional hazards models were performed to estimate the effect of anemia on death.

**Results:**

Of 436,658 patients at the baseline of ART initiation, the overall prevalence of anemia was 28.6%. During a median 2.65 (IQR: 1.80–3.51) years of follow-up after ART initiation, 376,325 (86.2%) patients had at least one Hb measurement (a total of 955,300 hemoglobin measurements). The annual prevalence of anemia after ART was 17.0%, 14.1%, 13.4%, 12.6% and 12.7%, respectively. Being anemic at the baseline of ART initiation (adjusted odds ratio, aOR = 6.80, 95% confidence interval (CI): 6.67–6.92) was the strongest factor associated with anemia after ART. Anemia status after ART showed a strong association with death after multivariable adjustment (mild anemia: adjusted hazard ratio (aHR) = 2.65, 95% CI: 2.55–2.76; moderate anemia: aHR = 4.60; 95% CI:4.40–4.81; severe anemia: aHR = 6.41; 95% CI:5.94–6.91).

**Conclusions:**

In the era of ART universal access, pre-ART anemia was common among HIV-infected patients. Notably, a certain proportion of anemia still persisted after ART, and was significantly associated with death. We recommend strengthening the monitoring of patients at risk of anemia, especially in patients with baseline anemia or during the first year of ART, and timely treatment for correcting anemia.

## Introduction

Anemia has always been a global public health issue, affecting nearly 23% of the world’s population and accounting for approximately 50.3 million healthy life years lost according to the Global Burden of Disease 2019 statistics [1]. Notably, anemia is of particular concern in HIV-infected patients. HIV-infected patients experience a higher risk of anemia than the general population [2]. Anemia, as one of the most common complications in HIV-infected patients [3, 4], is considered to be a predictor of poor health outcomes such as apparent fatigue, poor quality of life, rapid disease progression, and increased mortality [5].

The causes of anemia in HIV-infected patients are multifactorial, including HIV infection, aging, poor nutritional status, advanced disease, lower CD4^+^ T cell count as well as antiretroviral drugs [6, 7]. Common mechanisms of suffering from anemia in the general population such as nutritional deficiencies (including iron, vitamin B12 and folate), endemic anemia as well as infections including malaria and tuberculosis also occur in HIV-infected patients [8, 9]. Although antiretroviral therapy (ART) has been generally recognized as an effective treatment for improving anemia, several antiretroviral drugs are associated with anemia. Zidovudine (AZT), as one of the most critical components in the early history of ART, has been dramatically reduced in use due to its hematologic toxicity by bone marrow suppression [7]. Recent studies demonstrate that integrase strand transfer inhibitor (INSTI)-based regimens may also increase the risk of anemia [10, 11]. Of note, the selection of antiretroviral drugs is influenced not only by toxicity but also by complex factors such as drug availability, economic status, indications and prescribing physicians [12].

In the era of ART universal access, HIV-infected patients still face a higher burden of anemia. Recent findings reported a global prevalence of anemia among HIV-infected adults at 46.6% [12], at 19–33% in developed countries [11, 13, 14] and 32–67% in developing countries [15–18]. The prevalence of anemia varies markedly across different socioeconomic and clinical settings [12, 19]. A study among ART-naïve patients showed that the prevalence of anemia was 30.5% among patients with CD4^+^ T cell count < 200 cells/μL, and 60.1% among patients aged ≥ 60 years [20]. Currently, the increasing aging patients and the high proportion of late diagnosis among HIV-infected patients might contribute to the anemia burden [21, 22]. In addition, another study from North America showed that the prevalence of anemia among patients receiving ART declined from 33% to 2007 to 20% in 2017 [11]. Thus, universal access to ART and the use of AZT free treatment combination may partially alleviate the burden of anemia [23].

In China, the total number of patients living with HIV diagnosed in 2022 reached 1223 thousand since the first AIDS case was reported in 1985 [24]. The Chinese criterion for being eligible for ART initiation has been adjusted several times from CD4 ≤ 200 cells/μL in 2004, and revised to regardless of CD4 + T cell count in 2016. By the end of 2022, ART coverage achieved 92.8% [24]. Only few small studies from early ART era reported the prevalence of anemia among Chinese HIV-infected patients, ranging from 9.8 to 51.9% [20, 25, 26]. So far nationwide cohort studies on the prevalence of anemia are lacking in the Treat-all era in China. In addition, previous studies only confirmed the association between baseline anemia and mortality [25, 27], despite the fact that anemia during ART is time-dependent and may influence the association. The aim of this study is to evaluate the burden of anemia before and after ART and the impact of time-dependent anemia on outcomes in the national ART program.

## Methods

### Study design and participants

**Fig. 1:**
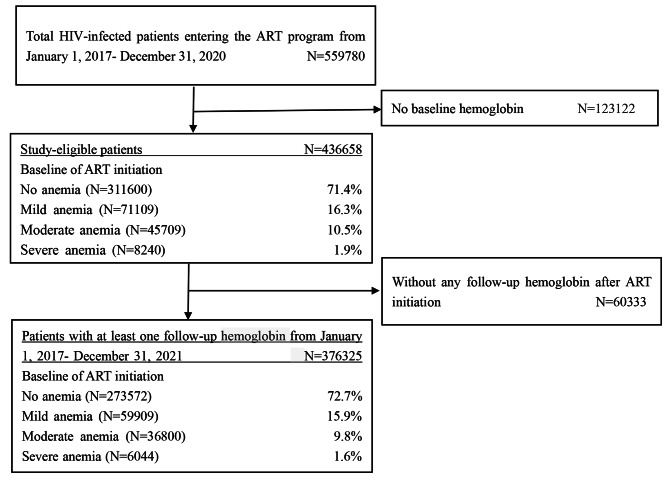
Flowchart of the study cohort Abbreviation: ART: antiretroviral therapy

We conducted an observational longitudinal cohort study among HIV-infected patients throughout the country who newly initiated ART from January 1, 2017 to December 31, 2020 in China (Fig. 1). The follow-up endpoint of this study was December 31, 2021. Eligible patients were aged 15 years or older and had hemoglobin (Hb) measurements at the ART initiation. In the analysis of ART follow-up section, we selected those with at least one follow-up Hb measurement during the study observation period. This study was performed in accordance with the Declaration of Helsinki. This study was reviewed and approved by the Institutional Review Board of the National Center for AIDS/STD Control and Prevention, Chinese Center for Disease Control and Prevention. Since all data were de-identified and provided in the aggregated form, the informed consent was waived.

### Data resources

All data we used was retrieved from the Chinese National Free ART Program (NFATP) database, which has been described previously [28]. This database is a web-based ART data collection system, containing related information on all HIV-infected patients receiving ART in mainland China. The information collected includes socio-demographic, clinical, and laboratory data. CD4^+^ T cell count and routine laboratory tests including routine blood tests, kidney and liver function were required to test at ART baseline. Routine laboratory test was repeated by the clinician as needed during the treatment follow-up. CD4^+^ T cell count and viral load were provided every year after ART initiation. Clinical visits were conducted at 0.5, 1, 2, 3 months after ART initiation and every 3 months thereafter.

### Definitions of outcomes and other variables

Primary outcomes were the prevalence of anemia at the baseline of ART initiation and after ART. Secondary outcomes were the effect of anemia on death after ART. When assessing the annual prevalence of anemia after ART, the last Hb test was selected if two or more measurements were available each year. Anemia was defined as Hb level less than 13 g/dL for males or less than 12 g/dL for females according to the World Health Organization criteria [29]. It was further classified into mild anemia (11–12.9 g/dL in males or 11–11.9 g/dL in females), moderate anemia (8–10.9 g/dL), and severe anemia (< 8 g/dL).

Body mass index (BMI) scores were generated by the formula of (weight in kilograms)/(height in meters) [2]. Estimated glomerular filtration rate (eGFR) was generated by the Modification of Diet and in Renal Disease (MDRD) equation, eGFR = 186×serum creatinine (mg/dL) − 1.154 × [age(years)] − 0.203 × [0.742 if female] × [1.212 if black]. OIs were defined according to the Chinese Diagnosis criteria for HIV/AIDS [30]. Detailed OI data at ART baseline were only available from the designated hospitals/clinics. Based on available antiretroviral drugs in China, ART regimen was categorized into non-nucleoside reverse transcriptase inhibitor (NNRTI), protease inhibitor (PI), INSTI, and abnormal prescription or missing. Depending on the use of NRTI, the backbone of ART was divided into five groups, tenofovir disoproxil fumarate (TDF), AZT, abacavir (ABC), tenofovir alafenamide (TAF) and abnormal prescription or missing. The 2016 version of Chinese free ART guideline recommended TDF or AZT + Lamivudine + Efavirenz as the first-line regimen, with TDF preferred over AZT. And AZT should not be used in HIV-infected patients with Hb less than 90 g/L [31]. Financially affordable patients could access drugs beyond the scope of the free catalog. Patients with specific conditions such as severe anemia, hepatic or renal abnormalities, or severe opportunistic infections are recommended to delay initiation of ART until their condition improves [32].

### Statistical analyses

Continuous variables were described with median and interquartile ranges (IQR) or mean ± standard deviation. Categorical variables were presented as frequencies and proportions. The baseline characteristics of the cohort were described, stratified by the magnitude of anemia at the baseline of ART initiation. Then the annual prevalence of anemia after ART initiation was calculated.

Generalized estimating equation models (GEE) were used to explore the factors associated with anemia after ART initiation. The dependent variable was the status of anemia at each follow-up visit. Explanatory variables included fixed characteristics and time-varying variables. The fixed variables included age, gender, route of HIV transmission, BMI, eGFR and OIs at ART initiation. CD4^+^ T cell count, viral load, NRTI backbone, ART regimen and duration of ART were considered as time-dependent variables. Univariate and multivariate GEE analyses were performed on all covariates to calculate the odds ratio (OR) and 95% confidence interval (CI) for anemia. Time-varying Cox proportional hazards models were used to evaluate the effect of anemia on death. CD4^+^ T cell count, viral load, NRTI backbone, ART regimen and anemia status were considered as time-dependent variables. The observation time for each patient was calculated from the ART initiation to the date of death, dropout or the follow-up endpoint of this study, whichever came first. Two-sided *P*-values < 0.05 were considered statistically significant. All calculations were performed using SAS version 9.1 (SAS Institute, Cary, United States of America).

## Results

A total of 559,780 patients entered the NFATP from January 1, 2017, to December 31, 2020, 123,122 patients were excluded due to missing Hb measurement at ART initiation. We assessed the baseline homogeneity of included and excluded patients, and found the difference in baseline information was ± 5% (data not shown). Finally, 436,658 patients entered the study cohort (Fig. 1).

At the baseline of ART initiation, the overall prevalence of anemia in the national cohort was 28.6%, with mild, moderate, and severe anemia for 16.3%, 10.5%, and 1.9%, respectively. As shown in Table 1, the proportion of anemia was higher in concurrent OIs group, the older age group, the lower baseline eGFR, BMI or CD4^+^ T cell count group. In patients with the concurrent OIs, baseline eGFR < 60ml/min/1.73m^2^, baseline BMI < 18.5 kg/m^2^, baseline CD4^+^ T cell count < 200 cells/μL or age ≥ 50 years, the proportion of total anemia was 61.4%, 58.2%, 50.6%, 47.8% and 41.6%, respectively.

**Table 1:** Baseline characteristics of HIV-infected patients at ART initiation from 2017–2020, stratified by the magnitude of anemia

	Total, N (% )	Total Anemia, N (%)	Magnitude of Anemia		
			Mild anemia N (% )	Moderate anemia N (% )	Severe anemia N (% )
Overall	436,658	125,058 (28.6)	71,109 (16.3)	45,709 (10.5)	8240 (1.9)
**Age, years**					
15–24	46,615 (10.7)	5642 (12.1)	3424 (7.3)	1849 (4.0)	369 (0.8)
25–49	226,183 (51.8)	51,213 (22.6)	27,764 (12.3)	19,246 (8.5)	4203 (1.9)
≥ 50	163,860 (37.5)	68,203 (41.6)	39,921 (24.4)	24,614 (15.0)	3668 (2.2)
**Sex**					
Male	335,735 (76.9)	88,624 (26.4)	54,553 (16.2)	28,356 (8.4)	5715 (1.7)
Female	100,923 (23.1)	36,434 (36.1)	16,556 (16.4)	17,353 (17.2)	2525 (2.5)
**Route of HIV transmission**					
Homosexual contact	112,358 (25.7)	16,761 (14.9)	10,934 (9.7)	4734 (4.2)	1093 (1.0)
Heterosexual contact	309,228 (70.8)	104,928 (33.9)	58,216 (18.8)	39,841 (12.9)	6871 (2.2)
Injecting drug use	14,168 (3.2)	3043 (21.5)	1801 (12.7)	985 (7.0)	257 (1.8)
Others/Missing	904 (0.2)	326 (36.1)	158 (17.5)	149 (16.5)	19 (2.1)
**Baseline BMI, kg/m** ^**2**^					
≥ 25	53,961 (12.4)	7963 (14.8)	5264 (9.8)	2358 (4.4)	341 (0.6)
18.5–24.9	257,021 (58.9)	70,547 (27.4)	42,373 (16.5)	24,251 (9.4)	3923 (1.5)
< 18.5	45,652 (10.5)	23,089 (50.6)	9949 (21.8)	10,577 (23.2)	2563 (5.6)
Missing	80,024 (18.3)	23,459 (29.3)	13,523 (16.9)	8523 (10.7)	1413 (1.8)
**Baseline eGFR, ml/min/1.73m** ^**2**^					
≥ 90	325,393 (74.5)	85,283 (26.2)	48,995 (15.1)	30,870 (9.5)	5418 (1.7)
60–89	79,645 (18.2)	26,697 (33.5)	15,611 (19.6)	9553 (12.0)	1533 (1.9)
< 60	11,163 (2.6)	6500 (58.2)	2727 (24.4)	2928 (26.2)	845 (7.6)
Missing	20,457 (4.7)	6578 (32.2)	3776 (18.5)	2358 (11.5)	444 (2.2)
**Baseline CD4** ^**+**^ **T cell count, cells/μL**					
≥ 500	54,810 (12.6)	7011 (12.8)	4637 (8.5)	2029 (3.7)	345 (0.6)
350–499	76,838 (17.6)	11,680 (15.2)	8000 (10.4)	3223 (4.2)	457 (0.6)
200–349	127,274 (29.1)	26,538 (20.9)	17,732 (13.9)	7830 (6.2)	976 (0.8)
< 200	148,973 (34.1)	71,261 (47.8)	35,781 (24.0)	29,567 (19.8)	5913 (4.0)
Missing	28,763 (6.6)	8568 (29.8)	4959 (17.2)	3060 (10.6)	549 (1.9)
**Baseline OIs**					
No	399,867 (91.6)	102,463 (25.6)	61,253 (15.3)	35,511 (8.9)	5699 (1.4)
Yes	36,791 (8.4)	22,595 (61.4)	9856 (26.8)	10,198 (27.7)	2541 (6.9)

Abbreviation: ART, antiretroviral therapy; BMI, body mass index, eGFR, estimated glomerular filtration rate; OIs, opportunistic infections

### Anemia after ART initiation

**Fig. 2:**
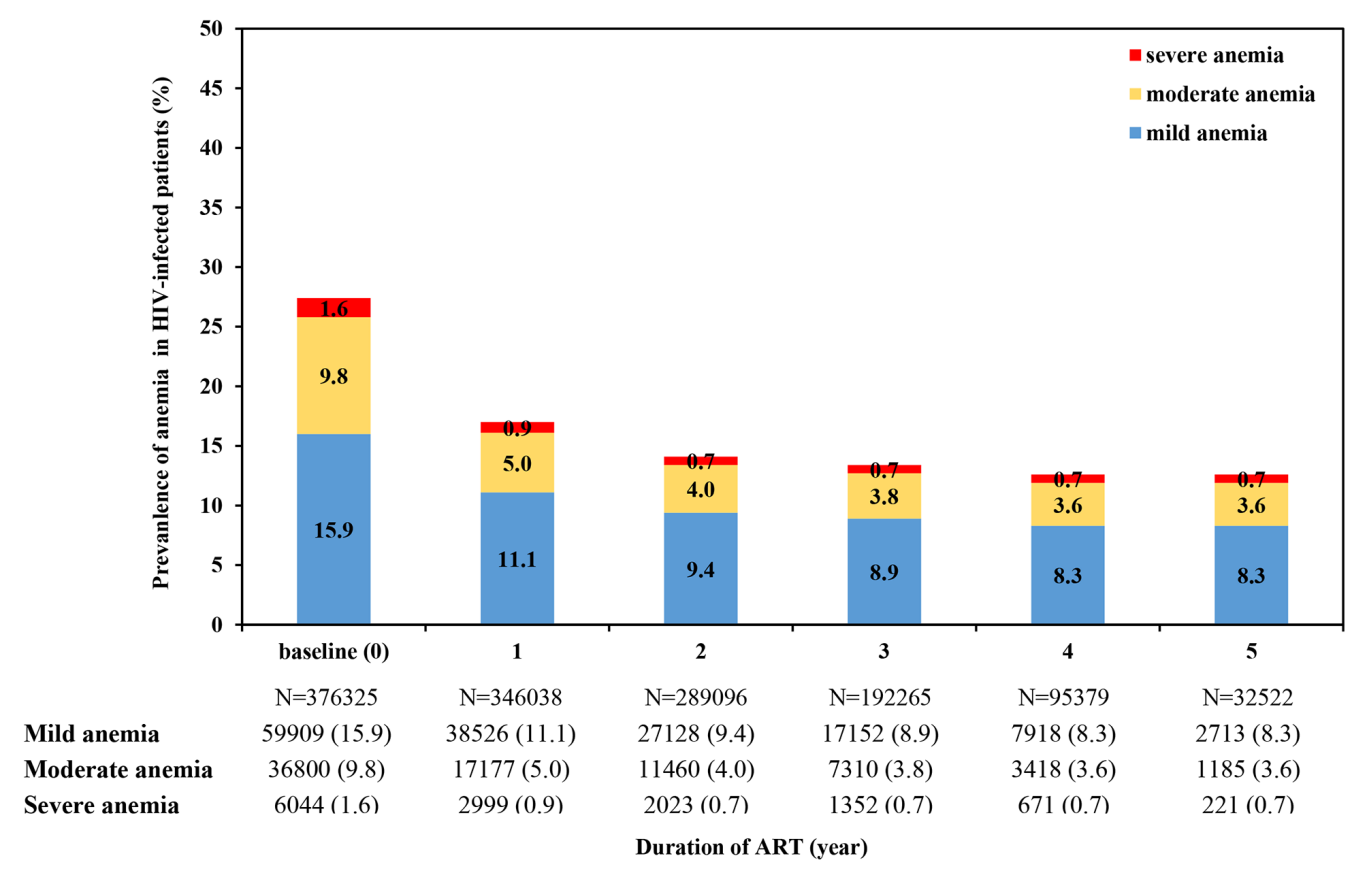
Prevalence of anemia before and after ART initiation among HIV-infected patients Abbreviation: ART, antiretroviral therapy

A total of 376,325 (86.2%) patients who had at least one Hb follow-up were included in the analysis (Fig. 1), with a median follow-up time was 2.65 (IQR: 1.80–3.51) years. 955,330 Hb measurements were available and the mean frequency of Hb testing after ART initiation was 2.06 ± 1.09. The annual prevalence of anemia was 17.0%, 14.1%, 13.4%, 12.6% and 12.7% from the first year to the fifth year after ART (Fig. 2).

In multivariate GEE model analysis (Table 2), being anemic at the baseline of ART initiation was the strongest factor associated with the increased odds of anemia during the ART follow-up (aOR = 6.80, 95% CI: 6.67–6.92). Compared with NNRTI-based regimen, the use of INSTI decreased the risk of anemia (aOR = 0.78, 95% CI: 0.74–0.83), but PI-based regimen (aOR = 1.43, 95% CI: 1.39–1.46) was more likely to have anemia after ART. The use of ABC (aOR = 1.47, 95% CI: 1.37–1.57) or AZT (aOR = 2.25, 95% CI: 2.19–2.30) had a significantly higher risk of anemia than TDF-contained regimen. Other factors including older age, female, acquired HIV through heterosexual contact or injecting drug use, low BMI, low eGFR, low CD4^+^ T cell count and high viral load were independent predictors of anemia.

**Table 2:** Factors associated with anemia among HIV-infected patients after ART initiation

	Number of Hb tests	Anemia (%)	Unadjusted OR (95%CI )	*P* value	Adjusted OR (95%CI )	*P* value
Overall	955,300	141,253				
**Baseline anemia**						
No	700,053	46,759 (6.68)	Ref		Ref	
Yes	255,247	94,494 (37.02)	8.45 (8.31,8.59)	< 0.001	6.80 (6.67,6.92)	< 0.001
**Age, years**						
15–24	109,410	7037 (6.43)	Ref		Ref	
25–49	516,026	56,501 (10.95)	1.81 (1.75,1.88)	< 0.001	1.15 (1.11,1.20)	< 0.001
≥ 50	329,864	77,715 (23.56)	4.57 (4.42,4.73)	< 0.001	1.77 (1.71,1.84)	< 0.001
**Sex**						
Male	732,645	86,778 (11.84)	Ref		Ref	
Female	222,655	54,475 (24.47)	2.23 (2.20,2.27)	< 0.001	1.70 (1.67,1.73)	< 0.001
**Route of HIV transmission**						
Homosexual contact	270,000	13,863 (5.13)	Ref		Ref	
Heterosexual contact	656,926	123,550 (18.81)	4.12 (4.02,4.22)	< 0.001	1.84 (1.79,1.89)	< 0.001
Injecting drug use	26,610	3530 (13.27)	2.69 (2.56,2.83)	< 0.001	2.00 (1.89,2.11)	< 0.001
Others/Missing	1764	310 (17.57)	3.81 (3.25,4.47)	< 0.001	1.71 (1.42,2.06)	< 0.001
**Baseline BMI, kg/m** ^**2**^						
≥ 25	119,223	12,105 (10.15)	Ref		Ref	
18.5–24.9	559,317	81,301 (14.54)	1.53 (1.49,1.58)	< 0.001	1.18 (1.15,1.22)	< 0.001
< 18.5	96,557	20,462 (21.19)	2.52 (2.44,2.61)	< 0.001	1.34 (1.29,1.39)	< 0.001
Missing	180,203	27,385 (15.2)	1.65 (1.60,1.71)	< 0.001	1.21 (1.17,1.25)	< 0.001
**Baseline eGFR, ml/min/1.73m** ^**2**^						
≥ 90	711,067	90,092 (12.67)	Ref		Ref	
60–89	171,379	34,344 (20.04)	1.72 (1.69,1.76)	< 0.001	1.26 (1.23,1.29)	< 0.001
< 60	21,364	9067 (42.44)	5.19 (5.00,5.40)	< 0.001	2.23 (2.13,2.33)	< 0.001
Missing	51,490	7750 (15.05)	1.28 (1.23,1.32)	< 0.001	1.09 (1.05,1.13)	< 0.001
**Baseline OIs**						
No	872,116	123,627 (14.18)	Ref		Ref	
Yes	83,184	17,626 (21.19)	1.69 (1.65,1.73)	< 0.001	0.78 (0.76,0.80)	< 0.001
**CD4** ^**+**^ **T cell count, cells/μL** ^**a**^						
≥ 500	256,608	24,155 (9.41)	Ref		Ref	
350–499	176,279	21,825 (12.38)	1.19 (1.17,1.21)	< 0.001	1.03 (1.01,1.05)	0.001
200–349	178,721	29,851 (16.7)	1.50 (1.47,1.52)	< 0.001	1.09 (1.07,1.11)	< 0.001
< 200	104,791	26,370 (25.16)	2.22 (2.18,2.26)	< 0.001	1.36 (1.33,1.39)	< 0.001
Missing	238,901	39,052 (16.35)	1.42 (1.41,1.44)	< 0.001	1.16 (1.13,1.19)	< 0.001
**Viral load, copies/mL** ^**a**^						
< 50	606,023	80,574 (13.3)	Ref		Ref	
50–999	52,158	8975 (17.21)	1.20 (1.17,1.22)	< 0.001	1.02 (1,00,1.05)	0.060
1000–99,999	26,921	5726 (21.27)	1.31 (1.27,1.35)	< 0.001	1.22 (1.18,1.26)	< 0.001
≥ 100,000	7057	2758 (39.08)	2.26 (2.13,2.39)	< 0.001	1.97 (1.86,2.10)	< 0.001
Missing	263,141	43,220 (16.42)	1.11 (1.10,1.12)	< 0.001	1.23 (1.20,1.25)	< 0.001
**NRTI Backbone** ^**a**^						
TDF	121,625	26,785 (22.02)	Ref		Ref	
AZT	788,969	104,017 (13.18)	1.91 (1.87,1.95)	< 0.001	2.25 (2.19,2.30)	< 0.001
ABC	9955	2868 (28.81)	2.21 (2.08,2.34)	< 0.001	1.47 (1.37,1.57)	< 0.001
TAF	17,864	1354 (7.58)	0.68 (0.65,0.71)	< 0.001	1.01 (0.94,1.09)	0.782
Abnormal prescription/Missing	16,887	6229 (36.89)	2.52 (2.42,2.62)	< 0.001	1.72 (1.63,1.81)	< 0.001
**ART regimen** ^**a**^						
NNRTI-based	808,893	110,079 (13.61)	Ref		Ref	
PI-based	102,599	25,931 (25.27)	1.88 (1.84,1.92)	< 0.001	1.43 (1.39,1.46)	< 0.001
INSTI-based	39,463	4157 (10.53)	0.85 (0.82,0.88)	< 0.001	0.78 (0.74,0.83)	< 0.001
Abnormal prescription/Missing	4345	1086 (24.99)	1.78 (1.65,1.91)	< 0.001	1.20 (1.10,1.31)	< 0.001
**Duration of ART, year** ^**a**^						
4–5	32,522	4119 (12.67)	Ref		Ref	
3–4	95,379	12,007 (12.59)	1.01 (0.98,1.03)	0.496	1.15 (1.11,1.19)	< 0.001
2–3	192,265	25,814 (13.43)	1.07 (1.04,1.09)	< 0.001	1.31 (1.27,1.35)	< 0.001
1–2	289,096	40,611 (14.05)	1.10 (1.07,1.13)	< 0.001	1.42 (1.37,1.46)	< 0.001
0–1	346,038	58,702 (16.96)	1.31 (1.27,1.34)	< 0.001	1.81 (1.75,1.88)	< 0.001

^a^ CD4^+^ T cell count, viral load, NRTI backbone, ART regimen and duration after ART were time-updated variables

Abbreviation: ART, antiretroviral therapy; OR, odds ratio; CI, confidence interval; BMI, body mass index; eGFR, estimated glomerular filtration rate; OIs, opportunistic infections; TDF, tenofovir disoproxil fumarate; AZT, zidovudine; ABC, abacavir; TAF, tenofovir alafenamide; NNRTI, non-nucleoside reverse transcriptase inhibitor; PI, protease inhibitor; INSTI, integrase strand transfer inhibitor

### Anemia and mortality

During the follow-up period, 15,562 deaths were identified and the overall mortality rate was 1.52/100 person-years (PY). The mortality rate was 0.82/100PY in those who were not anemic. Stratified by the magnitude of anemia, the mortality rate was 3.40,5.45,8.17/100PY in those with mild, moderate and severe anemia. After adjusting for confounders in the time-varying Cox proportional hazards model, the severity of anemia was the strongest predictor for mortality. Compared with non-anemic patients, mild anemia (aHR = 2.65; 95% CI: 2.55–2.76), moderate anemia (aHR = 4.60; 95% CI:4.40–4.81), and severe anemia (aHR = 6.41; 95% CI:5.94–6.91) were strongly increased the association with mortality (Table 3).

**Table 3:** Factors associated with death among HIV-infected patients after ART initiation

	Death, N	Mortality rate per 100 PY	Unadjusted HR (95%CI)	*P* value	Adjusted HR (95%CI)	*P* value
Overall	15,562	1.52				
**Anemia status** ^**a**^						
No	6746	0.82	Ref		Ref	
Mild anemia	4363	3.40	4.31 (4.14,4.47)	< 0.001	2.65 (2.55,2.76)	< 0.001
Moderate anemia	3614	5.45	7.14 (6.85,7.44)	< 0.001	4.60 (4.40,4.81)	< 0.001
Sevre anemia	839	8.17	10.83 (10.07,11.64)	< 0.001	6.41 (5.94,6.91)	< 0.001
**Age, years**						
15–24	327	0.28	Ref		Ref	
25–49	4896	0.88	3.19 (2.85,3.56)	< 0.001	2.16 (1.93,2.42)	< 0.001
≥ 50	10,339	2.94	10.59 (9.48,11.82)	< 0.001	4.44 (3.96,4.98)	< 0.001
**Sex**						
Male	12,835	1.64	Ref		Ref	
Female	2727	1.14	0.69 (0.66,0.72)	< 0.001	0.49 (0.47,0.51)	< 0.001
**Route of HIV transmission**						
Homosexual contact	1294	0.46	Ref		Ref	
Heterosexual contact	13,617	1.93	4.23 (3.99,4.48)	< 0.001	1.90 (1.79,2.02)	< 0.001
Injecting drug use	629	1.93	4.21 (3.83,4.64)	< 0.001	2.60 (2.36,2.87)	< 0.001
Others/Missing	22	1.11	2.44 (1.60,3.71)	< 0.001	1.49 (0.98,2.27)	0.036
**Baseline BMI, kg/m** ^**2**^						
≥ 25	1122	0.88	Ref		Ref	
18.5–24.9	8583	1.43	1.62 (1.53,1.73)	< 0.001	1.22 (1.15,1.30)	< 0.001
< 18.5	2791	2.74	3.12 (2.91,3.35)	< 0.001	1.70 (1.58,1.82)	< 0.001
Missing	3066	1.58	1.80 (1.68,1.92)	< 0.001	1.23 (1.15,1.32)	< 0.001
**Baseline eGFR, ml/min/1.73m** ^**2**^						
≥ 90	9491	1.25	Ref		Ref	
60–89	3992	2.17	1.74 (1.67,1.80)	< 0.001	1.15 (1.11,1.20)	< 0.001
< 60	1205	5.23	4.19 (3.95,4.45)	< 0.001	1.52 (1.43,1.63)	< 0.001
Missing	874	1.52	1.25 (1.16,1.34)	< 0.001	1.22 (1.13,1.30)	< 0.001
**Baseline OIs**						
No	12,930	1.38	Ref		Ref	
Yes	2632	3.08	2.25 (2.15,2.34)	< 0.001	1.29 (1.24,1.35)	< 0.001
**CD4** ^**+**^ **T cell count, cells/μL** ^**a**^						
≥ 500	691	0.24	Ref		Ref	
350–499	868	0.43	1.79 (1.62,1.98)	< 0.001	1.33 (1.20,1.47)	< 0.001
200–349	1570	0.76	3.13 (2.86,3.42)	< 0.001	1.62 (1.48,1.77)	< 0.001
< 200	2790	2.29	9.23 (8.49,10.04)	< 0.001	2.82 (2.59,3.08)	< 0.001
Missing	9643	4.80	22.68 (20.99,24.51)	< 0.001	4.18 (3.83,4.56)	< 0.001
**Viral load, copies/mL** ^**a**^						
< 50	3279	0.47	Ref		Ref	
50–999	590	0.96	1.96 (1.79,2.14)	< 0.001	1.37 (1.26,1.50)	< 0.001
1000–99,999	675	1.98	4.11 (3.78,4.46)	< 0.001	2.72 (2.51,2.96)	< 0.001
≥ 100,000	583	6.56	13.4 (12.27,14.63)	< 0.001	5.35 (4.89,5.86)	< 0.001
Missing	10,435	4.65	11.39 (10.94,11.85)	< 0.001	4.76 (4.50,5.04)	< 0.001
**NRTI Backbone** ^**a**^						
TDF	12,632	1.47	Ref		Ref	
AZT	2153	1.58	1.09 (1.04,1.14)	< 0.001	1.04 (0.99,1.09)	0.132
ABC	260	3.21	2.18 (1.93,2.46)	< 0.001	1.03 (0.90,1.17)	0.690
TAF	87	0.85	0.58 (0.47,0.71)	< 0.001	0.74 (0.59,0.94)	0.014
Abnormal prescription/Missing	430	4.04	2.76 (2.51,3.04)	< 0.001	0.95 (0.85,1.06)	0.364
**ART regimen** ^**a**^						
NNRTI-based	13,437	1.47	Ref		Ref	
PI-based	1635	2.10	1.43 (1.36,1.51)	< 0.001	1.04 (0.98,1.10)	0.191
INSTI-based	365	1.26	0.85 (0.77,0.94)	0.002	0.79 (0.70,0.89)	< 0.001
Abnormal prescription/Missing	125	3.03	2.05 (1.72,2.45)	< 0.001	1.29 (1.08,1.55)	0.005

^a^ CD4^+^ T cell count, viral load, NRTI backbone, ART regimen and anemia status were time-dependent variables

Abbreviation: ART, antiretroviral therapy; PY, person-years; HR, hazard ratio; CI, confidence interval; BMI, body mass index; eGFR, estimated glomerular filtration rate; OIs, opportunistic infections; TDF, tenofovir disoproxil fumarate; AZT, zidovudine; ABC, abacavir; TAF, tenofovir alafenamide; NNRTI, non-nucleoside reverse transcriptase inhibitor; PI, protease inhibitor; INSTI, integrase strand transfer inhibitor

## Discussion

This is the first study of a nationwide ART cohort to assess the burden of anemia in HIV-infected patients in China during the current ART era. We observed that pre-ART anemia was common, but severe anemia was infrequent (1.9%). Although anemia was improved after ART initiation, a certain percentage of patients were still anemic and baseline anemia was the strongest risk factor. Furthermore, we found that anemia status after ART was a significant predictor of death. Thus, the issue of anemia in both ART-naïve and ART-experienced patients still needs to be taken seriously.

In China, the prevalence of anemia among the general population was 8.93% according to the Global Burden of Disease Study [32]. In our study, pre-ART anemia prevalence among HIV-infected patients was 28.6%, which is much higher than that in the general population. Comparable results have been documented in other countries, including those studies conducted in Brazil (33%) [14], Ethiopia (31.8%) [17] and India (25.5%) [33]. However, a much higher proportion of anemia was reported in Uganda (67.4%) [34] and South Africa (70.5%) [35]. Another study nested within the PEARLS cohort study conducted in nine countries in diverse geographic settings found significant differences in the frequency of anemia, such as Malawi (67%), Haiti (67%), Thailand (47%) and Peru (37%) [14]. The burden of anemia in resource-limited countries might be higher, possibly due to nutrient deficiencies, more frequent infectious diseases and limited medical resources [36].

Moreover, we found that anemia was more prevalent in patients with older age, poor nutritional and clinical status. Similarly, a study in Uganda and Zimbabwe found that HIV-infected patients greater than 50 years were 2.6 times more likely to be anemic than patients of reproductive age [19]. This may not only be related to an age-related increase in comorbidities, but also aging itself may increase hematopoietic stem cell resistance to erythropoietin and proinflammatory cytokine expression [37]. Furthermore, HIV infection may accentuate aging progress through mechanisms such as chronic immune activation and inflammatory responses [38]. Additionally, previous studies support our finding that anemia was common among patients with lower CD4^+^ T cell count, especially in patients with CD4^+^ T cell count < 200 cells/μL [39]. Evidence has reported that lower CD4^+^ T cell count is associated with poor immune recovery, active viral replication and advanced disease, which are all contributing factors to anemia [40, 41].

Consistent with previous evidence [11], we observed a remarkable reduction of anemia after ART initiation, especially in the first year. In our study, the proportion of anemia declined from 27.3 to 17.0% within 12 months of ART. Similar findings were observed in a separate study in Ethiopia that the prevalence of anemia decreased from 42.9% at baseline to 14.3% at 12 months after ART [42]. Based on data from 34 ART cohorts, a systematic review showed that Hb continued to increase after initiating non-AZT-containing ART, the most significant increase was seen in the first year, with Hb increasing by 2.0 g/dL at 12 months and by 2.5 g/dL at 36 months [23]. The improvement of anemia after ART might be explained through inhibiting HIV replication, promoting immune reconstitution, and reducing the occurrence of OIs [12].

Our study revealed similar results to other studies, that being anemic at ART initiation was the strongest factor associated with anemia after ART [23]. A study conducted in rural Tanzania observed that there were one-third were still anemic after 12 months of ART among patients with anemia at ART initiation [43]. The persistence of anemia in HIV-infected patients may possibly be due to multifactorial causes [8], such as inflammatory states, nutritional deficiencies, co-infection with other viruses and antiretroviral drugs. necessitating additional intervention in addition to ART to correct anemia. In addition, we found that the use of INSTI decreased the risk of anemia. However, a study in the US showed an inconsistent outcome that INSTI increased the risk of anemia by 26% compared to NNRTI, which was considered due to the fact that patients with failed viral suppression or worse clinical status are more likely to switch to an INSTI-based regimen [10]. Our study also showed that patients with higher viral load are more likely to be anemic. This is consistent with prior studies that not only HIV infection but also active HIV viral replication could increase the risk of anemia [8].

Our findings provided evidence that the severity of anemia after ART was a significant predictor of mortality in HIV-infected patients. A long-term cohort in China showed that compared to patients who were not anemic at baseline, mild and moderate anemia increased the hazard of death by 60% and severe anemia by 86% [44]. Another analysis from EuroSIDA quantified the effect of Hb on death in HIV-infected patients, then found that the risk of death tended to increase by 57% with 1 g/dL decrease in Hb [45]. Based on similar findings, emerging studies attempted to use baseline Hb to predict prognostic outcomes. A nested case-control study found that adding baseline Hb to a prognostic model containing both CD4^+^ T cell count and viral load significantly improved the accuracy in predicting the risk of death in HIV-infected patients [27].

Our study has some limitations. Firstly, we only included those who initiated ART. Patients with severe anemia are more likely to be excluded from initiating ART until their condition improves to meet the criteria for initiating ART, which may underestimate the burden of anemia. Secondly, potential selection bias may have been introduced. Some patients were excluded due to a lack of Hb measurement at baseline and follow-up, although we found differences in baseline characteristics between included and excluded subjects to be within ± 5%. Additionally, ART regimen that patients received was not randomly assigned. Baseline characteristics of patients receiving different ART regimens may have differed. Therefore, we used multivariate analysis to correct for the effects of potential bias. Finally, all the data in our study were drawn from regular record registers. Due to the limited information reported, some other potential influencing factors such as socio-economic status and lifestyle, were not introduced in this study. And types of anemia before and after ART could not be classified, which is essential for determining the etiology of the anemia and for guiding precise interventions. Further research is needed.

## Conclusions

The findings of our nationwide cohort study indicate that pre-ART anemia was common among HIV-infected patients during the Treat-All era. Despite a significant reduction in the prevalence of anemia after ART, a certain proportion of anemia still persisted. Moreover, time-dependent anemia was significantly associated with death. We recommend strengthening the monitoring of patients at risk of anemia, especially in patients with baseline anemia or during the first year of ART. Timely detection of anemia and treatment for correcting anemia during ART is critical to improving the burden of anemia and prognosis of patients living with HIV.

## Data Availability

The datasets used and/or analyzed during the current study were available from the corresponding author on reasonable request.

## List of abbreviations

ABC: Abacavir
aHR: Adjusted hazard ratio
aOR: Adjusted odds ratio
ART: Antiretroviral therapy
AZT: Zidovudine
BMI: Body mass index
CI: Confidence interval
eGFR: Estimated glomerular filtration rate
Hb: Hemoglobin
INSTI: Integrase strand transfer inhibitor
IQR: Interquartile ranges
NNRTI: Non-nucleoside reverse transcriptase inhibitor
OIs: Opportunistic infections
PI: Protease inhibitor
TAF: Tenofovir alafenamide
TDF: Tenofovir disoproxil fumarate
